# Identification of taxonomic changes in the fecal bacteriome associated with colorectal polyps and cancer: potential biomarkers for early diagnosis

**DOI:** 10.3389/fmicb.2023.1292490

**Published:** 2024-01-11

**Authors:** Beatriz Alessandra Rudi Grion, Paula Luize Camargos Fonseca, Rodrigo Bentes Kato, Glen Jasper Yupanqui García, Aline Bruna Martins Vaz, Beatriz Nafría Jiménez, Ainhoa Lapitz Dambolenea, Koldo Garcia-Etxebarria, Bertram Brenig, Vasco Azevedo, Luis Bujanda, Jesus M. Banales, Aristóteles Góes-Neto

**Affiliations:** ^1^Laboratory of Molecular and Computational Biology of Fungi, Institute of Biological Sciences, Department of Microbiology, Federal University of Minas Gerais, Belo Horizonte, Brazil; ^2^Integrative Biology Laboratory, Institute of Biological Sciences, Department of Genetics, Ecology, and Evolution, Federal University of Minas Gerais, Belo Horizonte, Brazil; ^3^Public Health Laboratories, Ministry of Health, Brasília, Brazil; ^4^Graduate Program in Bioinformatics, Federal University of Minas Gerais, Belo Horizonte, Brazil; ^5^Oswaldo Cruz Foundation (Fiocruz-MG), Minas Gerais, Brazil; ^6^Medical School, Universidade José do Rosário Vellano (UNIFENAS), Belo Horizonte, Brazil; ^7^Department of Liver and Gastrointestinal Diseases, Biodonostia Health Research Institute – Donostia University Hospital, Ikerbasque, San Sebastian, Spain; ^8^Institute of Veterinary Medicine, Burckhardtweg, University of Göttingen, Göttingen, Germany; ^9^Laboratory of Cellular and Molecular Genetics, Federal University of Minas Gerais, Belo Horizonte, Brazil; ^10^CIBERehd, Madrid, Spain; ^11^Department of Biochemistry and Genetics, University of Navarra, Pamplona, Spain

**Keywords:** colorectal cancer, gut microbiome, metagenomics, bacteriome, biomarkers

## Abstract

Colorectal cancer (CRC) commonly arises in individuals with premalignant colon lesions known as polyps, with both conditions being influenced by gut microbiota. Host-related factors and inherent characteristics of polyps and tumors may contribute to microbiome variability, potentially acting as confounding factors in the discovery of taxonomic biomarkers for both conditions. In this study we employed shotgun metagenomics to analyze the taxonomic diversity of bacteria present in fecal samples of 90 clinical subjects (comprising 30 CRC patients, 30 with polyps and 30 controls). Our findings revealed a decrease in taxonomic richness among individuals with polyps and CRC, with significant dissimilarities observed among the study groups. We identified significant alterations in the abundance of specific taxa associated with polyps (Streptococcaceae, *Lachnoclostridium*, and *Ralstonia*) and CRC (Lactobacillales, Clostridiaceae, *Desulfovibrio*, SFB, *Ruminococcus*, and *Faecalibacterium*). Clostridiaceae exhibited significantly lower abundance in the early stages of CRC. Additionally, our study revealed a positive co-occurrence among underrepresented genera in CRC, while demonstrating a negative co-occurrence between *Faecalibacterium* and *Desulfovibrio*, suggesting potential antagonistic relationships. Moreover, we observed variations in taxonomic richness and/or abundance within the polyp and CRC bacteriome linked to polyp size, tumor stage, dyslipidemia, diabetes with metformin use, sex, age, and family history of CRC. These findings provide potential new biomarkers to enhance early CRC diagnosis while also demonstrating how intrinsic host factors contribute to establishing a heterogeneous microbiome in patients with CRC and polyps.

## 1 Introduction

Colorectal cancer (CRC) is a significant global health concern, raking as the third most diagnosed cancer and the second deadliest cause of cancer-related deaths worldwide, affecting both sexes and contributing to approximately 10% of cancer-related deaths (Sung et al., 2021). Incidence rates of CRC are approximately four times higher in developed countries compared to developing countries undergoing transition (Sung et al., 2021), and there is a concerning upward trend in incidence rates in developing nations (Arnold et al., 2017). The prognosis and chances of survival for individuals with CRC largely depend on the stage of cancer at the time of diagnosis, with better outcomes associated with early detection (Maringe et al., 2013; Rawla et al., 2019). Consequently, public health agencies have placed emphasis on expanding CRC diagnostic screening programs (Patel et al., 2022), aiming to improve early detection rates and ultimately reduce mortality rates associated with CRC.

The early detection of CRC presents a significant challenge due to low participation rates in screening programs among screen-eligible adults, with approximately one-third of individuals not participating (Joseph et al., 2018). Additionally, it is concerning to note that one in four diagnosed patients already have advanced neoplasia (U.S. Cancer Statistics: Colorectal Cancer Stat Bite | CDC, 2022). CRC is a heterogeneous disease primarily attributed to distinct embryological origins of the right and left colon (Bufill, 1990) coupled with diverse genetic and epigenetic backgrounds (Grady and Carethers, 2008; Binefa et al., 2014; Petrelli et al., 2017). This heterogeneity leads to various pathways of carcinogenesis (Keum and Giovanucci, 2019), resulting in multiple molecular subtypes (Menter et al., 2019) and a wide range of clinical manifestations (Sawicki et al., 2021). While hereditary syndromes and family history account for approximately 35–40% of CRC cases, the majority of cases (60–65%) are sporadic and arise from acquired genomic aberrations (Keum and Giovanucci, 2019). Therefore, CRC is a multifactorial disease influenced by environmental factors.

The majority of sporadic CRC cases arise in patients with premalignant lesions known as polyps, with adenomas being the predominant subtype (85–90%), along with a smaller proportion of serrated polyps (10–15%) (Conteduca et al., 2013). Colorectal adenomatous polyps are present in nearly half of the population aged 60 and above (Levine and Ahnen, 2006), but only 10% of these polyps have the potential to develop CRC (Conteduca et al., 2013). Colonoscopy remains the gold standard for CRC diagnosis (Conteduca et al., 2013; 2014), despite being an invasive and costly procedure that limits accessibility (Issa and NouredDine, 2017). Moreover, identifying polyps with cancerous potential, particularly the serrated subtypes, poses challenges due to anatomical and histological variations (Conteduca et al., 2013; Abdeljawad et al., 2015). Alternative methods, such as fecal immunochemical test (FIT) for detecting occult blood in feces, offer better accessibility but have lower sensitivity for early-stage CRC (Elsafi et al., 2015). Enhancing the accessibility and sensitivity of diagnostic methods could facilitate the identification of asymptomatic individuals with CRC or high-risk polyps, potentially leading to improved prognosis and survival rates.

The presence of CRC is commonly associated with altered microbial diversity within the gut, characterized by increased abundance of pathogenic microorganisms and/or depletion of those considered protective or beneficial (Wong and Yu, 2019). However, the contribution of microorganisms found in fecal and mucosal samples to CRC pathogenesis has not been fully elucidated. It is plausible that CRC pathogenesis is influenced by microbial metabolism, the invasion of host cells and/or the modulation of host immune system by the microbiome (Ternes et al., 2020). Nonetheless, numerous studies have demonstrated an enrichment of certain bacteria associated with CRC, which can serve as useful biomarkers for diagnosis, prognosis, and potentially treatment (Ternes et al., 2020).

Metagenomic studies have expanded our understanding of the microbial diversity present in the tumor environment and its involvement with the development of colorectal polyps (Mira-Pascual et al., 2015; Peters et al., 2016; Yu et al., 2019). However, limited research has investigated the correlations between bacterial abundance and the occurrence of polyps and CRC, considering their subclassifications, patient lifestyle, and associated comorbidities (Song and Chan, 2019). In this study, we analyzed the taxonomic diversity of bacteria in fecal samples using shotgun sequencing, comparing the relative abundance of bacteria among individuals with polyps, CRC, and a control group.

## 2 Materials and methods

### 2.1 Data collection

The fecal samples were collected between 2017 and 2018 by the Biodonostia Health Research Institute – Donostia Hospital San Sebastián, Spain, following the 2003 European Guidelines and the 2006 National Strategy against Cancer. The population-based screening of CRC was approved by the Basque Autonomous Government and implemented in 2009. The screening is based on the detection of fecal occult blood (FOB) using a biennial FIT, targeting women and men between 50 and 69 years old and a colonoscopy under sedation for FIT positive cases. The FIT test used was OC-Sensor (Eiken Chemical Co. Tokyo, Japan). Only one sample was collected per patient, and the hemoglobin concentration cut-off (f-Hb) was 100 ng Hb/mL. All samples used had an f-Hb ≥ 20 μg hemoglobin/g of feces, which is the threshold used in Spain to request colonoscopy examination.

Ninety participants were recruited from the screening, all with an f-Hb ≥ 20 μg Hb/g of feces, who subsequently underwent a colonoscopy examination. Based on the colonoscopy results, the 90 individuals were divided into three groups: control group (*n* = 30; stool samples from individuals with colonoscopy showing no intestinal lesions, no history of diarrhea, and no history of previous intestinal infection), polyps group (*n* = 30; stool samples from individuals with colonoscopy showing polyps on the surface of the colon or rectum), and the CRC group (*n* = 30, which consists of individuals with biopsies confirming malignancy of colon or rectum lesions).

In addition to the exams, clinical information about each patient was also collected and made available for each sample such as: sex, weight, age, presence of comorbidities and genetic diseases, medication use, tobacco smoking and alcohol consumption, polyps histology, tumor locations and CRC staging (Tables 1–3).

**Table 1:** Clinical metadata from the individuals investigated in our study.

Variables	Control (*n* = 30)	Polyp (*n* = 30)	CRC (*n* = 30)
Male	9 (30%)	16 (53%)	21 (70%)
Female	21 (70%)	14 (47%)	9 (30%)
Mean age	59	62	64
Family history of CRC	4 (13%)	6 (20%)	7 (23%)
Hypertension	12 (40%)	14 (47%)	14 (47%)
Dyslipidemia	6 (20%)	15 (50%)	9 (30%)
Obesity	4 (13%)	10 (33%)	5 (17%)
Overweight + Obesity	18 (60%)	24 (80%)	16 (53%)
Diabetes	2 (7%)	0	4 (13%)
Hypothyroidism	4 (13%)	2 (7%)	2 (7%)
Hyperthyroidism	4 (13%)	0	1 (3%)
Depression/Anxiety	4 (13%)	6 (20%)	1 (3%)
Omeoprazole	7 (23%)	4 (13%)	5 (17%)
Calcium/Vitamin D	4 (13%)	2 (7%)	6 (20%)
Aspirin	2 (7%)	2 (7%)	2 (7%)
Alcohol consumption	12 (40%)	14 (47%)	16 (57%)
Smoking	5 (17%)	12 (40%)	7 (25%)
Former smoking	5 (17%)	5 (7%)	10 (36%)

**Table 2:** Characteristics of polyp group.

Histology	Polyp (*n* = 30)
Polyps ≥1 cm	24 (80%)
Adenoma	29 (97%)
Tubular	19 (65%)
(Tubular + Tubulovillous)	6 (21%)
Tubulovillous	4 (14%)
Villous	0
Serrated	14 (47%)
Hyperplastic	12 (40%)
Traditional serrated	1 (3%)
Sessile serrated	0

**Table 3:** Characteristics of CRC group.

Tumor location	CRC (*n* = 30)
Rectum	8 (29%)
Sigmoid colon	12 (43%)
Descending colon	3 (11%)
Splenic flexure	1 (3%)
Transverse colon	0 (0%)
Ascending colon	2 (7%)
Hepatic flexure	0 (0%)
Cecum	2 (7%)
Unknown location*	2 (7%)
Staging of CRC	
I	9 (50%)
II	5(28%)
III	2 (11%)
IV	2 (11%)
Unknown staging**	12 (40%)
Deaths from CRC	10 (33%)

### 2.2 Data processing

The samples were sent to the Laboratory of Molecular and Computational Biology of Fungi (LBMCF) for processing and sequencing. To optimize the extraction of DNA, the samples were homogenized in a vortex and lyophilized (Gudra et al., 2019). Total DNA was extracted with FastDNA kit (MP Biomedicals, CA, United States) according to the manufacturer’s instructions. Metagenomic DNA was run in a gel to check for integrity and quantified using Qubit dsDNA BR Assay Kit (Thermo Fisher Scientific, MA, United States).

Total metagenomic DNA was fragmented by standard shotgun sequencing. Libraries with an average fragment size of 450 bp were prepared from genomic DNA using the NEBNext Fast DNA Fragmentation and Library Preparation Kit (New England Biolabs, Ipswich, NE-USA) following the manufacturer’s instructions. The library quality was assessed using the Agilent 2,100 Bioanalyzer, and shotgun metagenomic sequencing was performed using an Illumina HiSeq 2,500 instrument (Illumina, CA, United States).

### 2.3 Data analysis

Overall quality of the reads was evaluated using FastQC vO.11.5 (Andrews, 2010). Adapters and low-quality sequences (Phred score < 20) were removed with BBtools (Bushnell, 2014). Reads that passed in quality check were mapped to the human genome (GCF_000001405) using Bowtie2 v2.4.2 (Langmead and Salzberg, 2012) to remove host DNA. Reads without mapping to human genome were used to identify Bacteria through MAPseq v1.2.6 (Matias Rodrigues et al., 2017). All the complete genomes of Bacteria found in the NCBI public database were used as reference, and Operational Taxonomic Units (OTUs) were classified using the complete 16S rRNA gene, with ≥97% identity threshold.

### 2.4 Statistical analysis

Statistical analyses of diversity and abundance were performed using the Microbiome Analyst platform (Dhariwal et al., 2017; Chong et al., 2020). Sequencing depth was evaluated using rarefaction curves for all samples. To explore bacterial taxonomic ranks, the relative abundance profiling was analyzed for all taxonomic levels. The community profile per sample was assessed through alpha and beta diversities, using Chao1 and Shannon diversity measures, and Bray-Curtis index, respectively. Additionally, a co-occurrence network was constructed based on statistically significant bacteria associated with each group. All OTUs with significant statistical abundance within groups were analyzed using classical univariate statistical comparisons (ANOVA) for all taxonomy levels. Subsequently, we identified predictive features (biomarkers) through Random Forests, a machine learning algorithm for classification. We utilized 5,000 trees and seven predictors for the classification of the three groups. Also, we have calculated accuracy and F1-score utilizing Weka (Frank et al., 2016), which are metrics for evaluation of models through Machine Learning based on the confusion matrix from our Random Forest analysis. To calculate these metrics, we first calculated the TPR (The True Positive Rate, also known as sensitivity or recall), FPR (False Positive Rate), TNR (True Negative Rate, or specificity), and FNR (False Negative Rates). Finally, we calculated the precision, the number of true positives divided by the number of true positives plus the number of false positives. These rates are important for evaluating the performance of our model in each class.

All clinical information regarding each patient, as well as information about polyp and CRC characteristics, were analyzed using the Chao1 and Shannon diversity indices, along with classical univariate statistical comparisons (ANOVA) for all taxonomic levels.

For all the above analysis in Microbiome Analyst, a low count filter was applied to the abundance table, considering only OTUs with a minimum of 2 counts and 20% prevalence in the samples. Low abundance OTUs were removed based on prevalence. Additionally, a low variance filter (20%) was applied based on the inter-quantile range (IQR) (10%). OTUs with low variance were removed based on the IQR. The number of OTUs remaining after the data filtering step was subsequently submitted to data transformation using the centered-log-ratio (CLR) transformation.

## 3 Results

### 3.1 The alpha diversity in the polyp and CRC groups is lower compared to control group

Shotgun metagenomic sequencing of all 90 samples yielded a total of 2,460,307,369 raw reads. Among them, 1,391,392 were not mapped to human genome corresponded to segments of 16S rDNA, which were subsequently assembled into operational taxonomic units (OTUs). The number of reads per sample ranged from a minimum of 6,491 to a maximum of 28,333 (Supplementary Tables S1, S2). In total, 8,610 OTUs related to bacteria were identified across all samples, with 7,590 OTUs composed of more than two reads. Among these OTUs, 6,619 low-abundance features were removed based on prevalence in less than 20% of the patients. Additionally, 98 low-variance features were removed based on interquartile range (IQR) calculations. After applying these filtering steps, a total of 874 OTUs remained. The rarefaction curves demonstrated sufficient sampling depth for all groups, with less than 1% singletons observed in all samples (Supplementary Figure S1).

**Figure 1:**
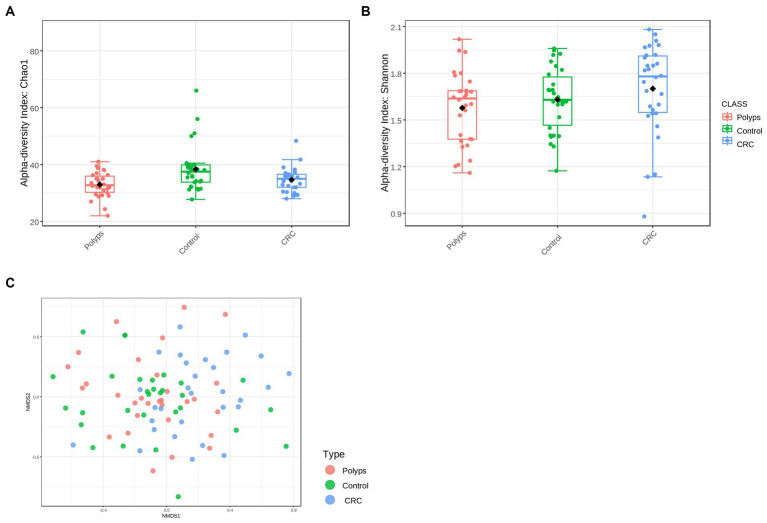
Alpha and beta diversity in the groups of patients with polyps, control, and CRC. **(A)** Alpha diversity at the Order level, using the Chao 1 index with a value of *p* = 0.0017615 and an *F*-value = 6.8271; the polyps and CRC groups exhibit lower diversity compared to the control group. **(B)** Alpha diversity at the order level, using the Shannon index, with a value of *p* = 0.15125 and an *F*-value = 1.9304; The CRC group tends to show higher alpha diversity. **(C)** The NMDS analysis revealed a significant separation in the diversity of the bacterial community (value of *p* = 0.001, *F*-value = 1.7416, *R*^2^ = 0.038496, NMDS Stress = 0.28604).

Alpha diversity is a measure of microbial diversity within a sample, taking into account both the richness (number of different species) and the abundance of operational taxonomic units (OTUs). In this study, we evaluated alpha diversity using two indices: the Chao1 index, which measures richness and values rare species, and the Shannon diversity index, which assesses both richness and abundance (Prehn-Kristensen et al., 2018). Based on the Chao1 index, we observed significantly different values among the groups at the order taxonomic level (value of *p* <0.00017615; ANOVA test, *F*-value = 6.8271) (Figure 1A). The polyps and CRC groups exhibited lower alpha diversity (median of 32.75 and 35.0, respectively) compared to the control group (median = 37.5). Regarding the Shannon diversity index, the CRC group showed a slight increase compared to the control group, but without statistically significant differences between the groups at any taxonomic level (value of *p* = 0.15125, *F*-value = 1.9304 for the order level) (Figure 1B).

To assess the variability of bacterial community among the three study groups, beta diversity was analyzed (Figure 1C). Non-metric multidimensional scaling (NMDS) analysis was employed to compare the similarities between the samples using the Bray-Curtis dissimilarity index. The significance of differences in beta diversity among the study groups was determined using permutational multivariate analysis of variance (PERMANOVA). The NMDS analysis revealed significant separation in bacteriome community diversity among groups at both the OTU level (value of *p* = 0.002; *F*-value = 1.5641; *R*^2^ = 0.034709; NMDS stress = 0.26666) and the genus level (value of *p* = 0.001; *F*-value = 1.7416; *R*^2^ = 0.038496; NMDS stress = 0.28604) (Figure 1C). Furthermore, there were high dissimilarities observed among the control, polyps, and CRC groups, but with some overlaps between the microbial communities. This indicates that the microbiomes of control individuals may exhibit some similarities to those of CRC patients, and conversely, some CRC patients may have a microbiome that is more akin to that of unaffected individuals.

### 3.2 The microbiome of all patient groups is predominantly composed by firmicutes, bacteroidetes, actinobacteria, and proteobacteria, with no significant differences in relative abundance at taxonomic level

**Figure 2:**
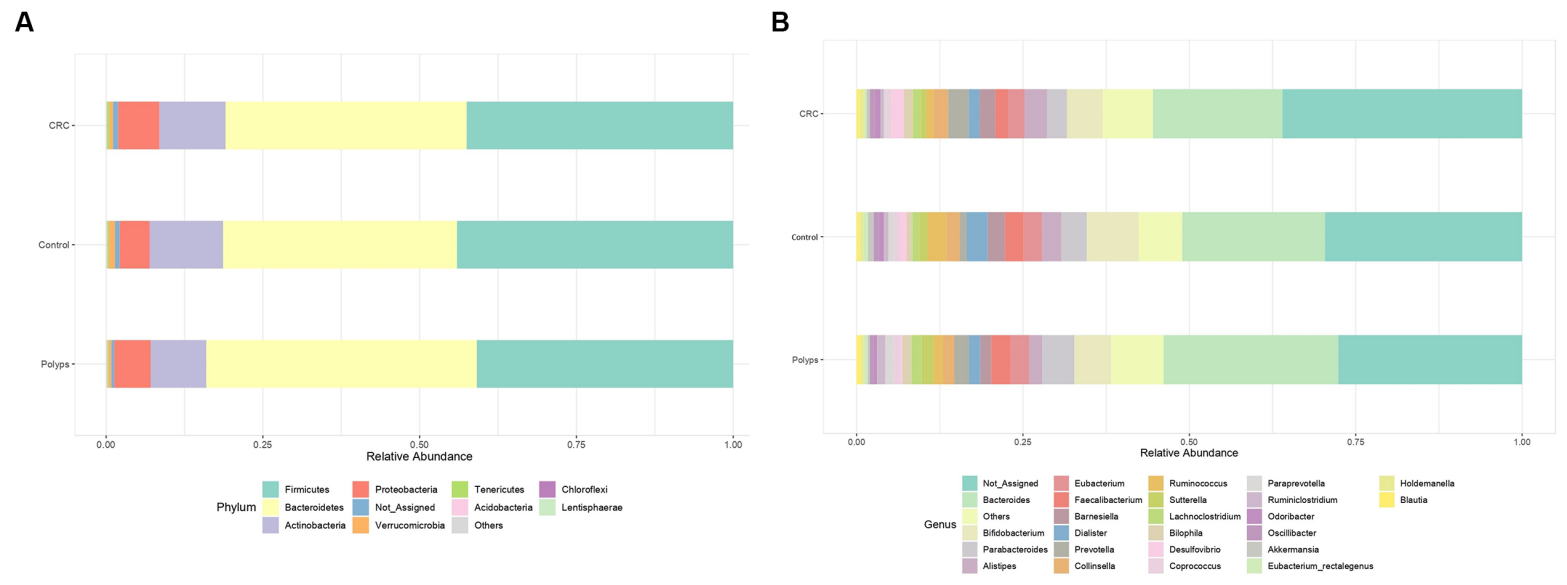
Relative abundance of bacteria in three groups. **(A)** Phylum level and **(B)** Genus level. At the phylum taxonomic level, the samples are predominantly dominated by Firmicutes, followed by Bacteroides, Actinobacteria, and Proteobacteria. At the genus taxonomic level *Bacteroides* is the most abundant genus in all three samples. The unassigned genera are moderately more abundant in CRC (37%) compared to control (30%), and polyps (28%).

We performed a visual exploration and analysis of the taxonomic composition in the CRC, polyps, and control groups, considering the relative abundance in percentage of microorganisms (Figure 2). At the phylum level, the samples are mainly dominated by Firmicutes, followed by Bacteroidetes, Actinobacteria, and Proteobacteria. No phylum showed a significantly differential abundance (Figure 2A). In terms of specific differences, the polyp group samples exhibited slightly higher levels of Bacteroidetes (42%) compared to the control group (36%) and CRC (37%). There was also a slight decrease in Actinobacteria abundance in the polyp group (8%) compared to the control group (11%) and CRC (10%). The abundance of Proteobacteria was marginally decreased in the control group (4.7%) compared to polyps (5.7%) and CRC (6.4%) groups. Furthermore, the Firmicutes to Bacteroidetes (F/B) ratio was higher in the control group compared to the CRC and polyp groups (Supplementary material).

### 3.3 Patients with polyps and CRC display notable differences in the relative abundance of bacterial taxa at the order, family, and genus levels

**Figure 3:**
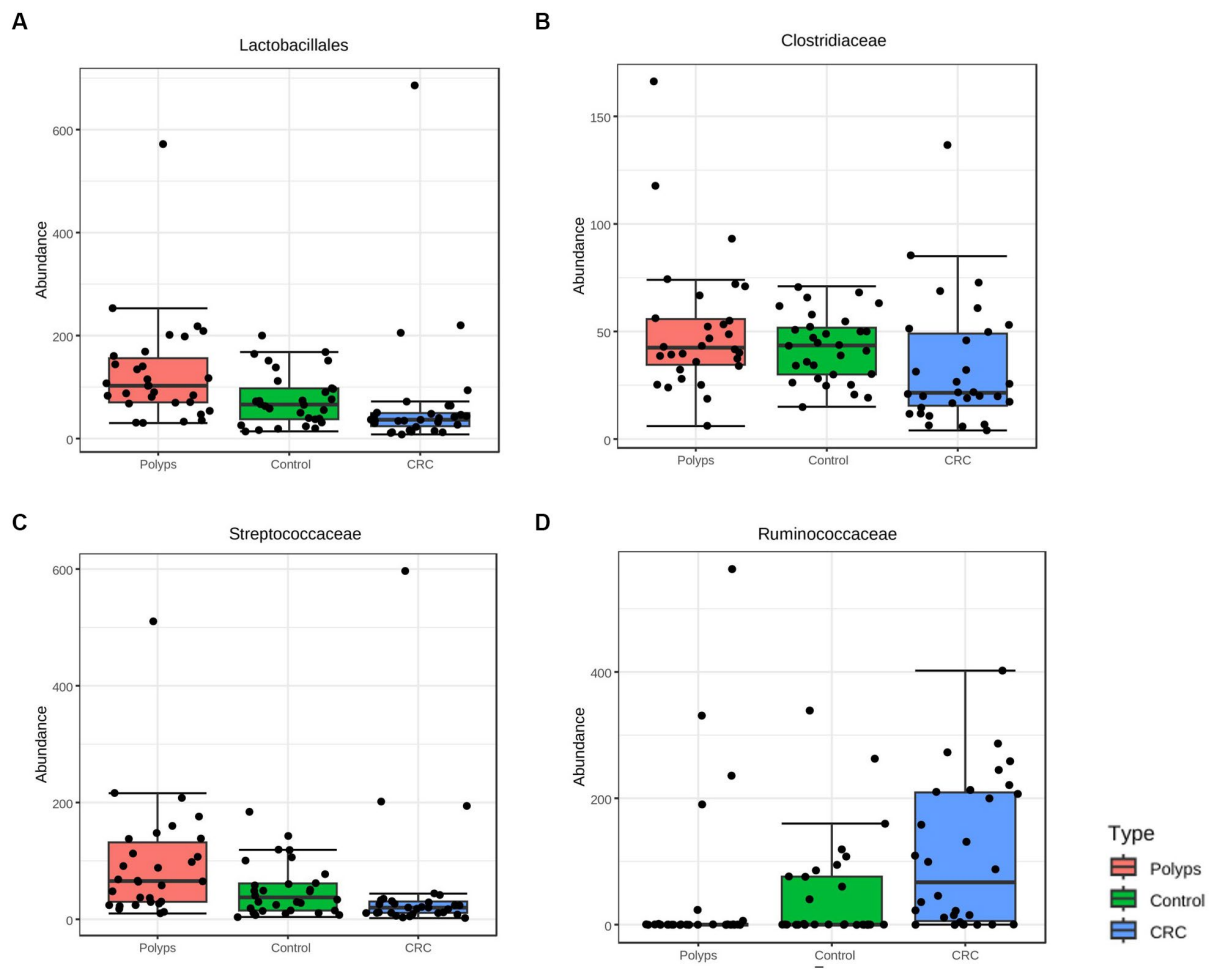
Taxa with significant differences in abundance among the three study groups at the taxonomic levels of Order and Family were identified using the ANOVA statistical method. **(A,B)** The abundance of the Order Lactobacillales (value of *p* = 0.0001; FDR = 0.007) and the Family Clostridiaceae (value of *p* = 0.005; FDR = 0.019) decreased in the CRC group compared to the control group. **(C)** The abundance of the Family Streptococcaceae increased in the samples of patients with polyps compared to the control group (value of *p* = 0.004; FDR = 0.019). **(D)** The abundance of the putative Family Ruminoccoccaceae (value of *p* = 0.001; FDR = 0.026) increased in the CRC group compared to the polyp and control groups.

At the taxonomic level of Order, there is a notable decrease in the relative abundance of Lactobacillales in CRC samples and an increase in the polyp group, compared to the control group (Figure 3A; value of *p* = 0.0001; FDR = 0.007). At the Family level, Clostridiaceae is less abundant in CRC patient samples (Figure 3B; value of *p* = 0.005; FDR = 0.019), while Streptococcaceae is more abundant in the polyp group and less abundant in the CRC group, compared to the control group (Figure 3C; value of *p* = 0.004; FDR = 0.019). Furthermore, the abundance of the putative family Ruminococcaceae is increased in the CRC group and decreased in the polyp group, compared to the control group (Figure 3D; value of *p* = 0.001; FDR = 0.026).

**Figure 4:**
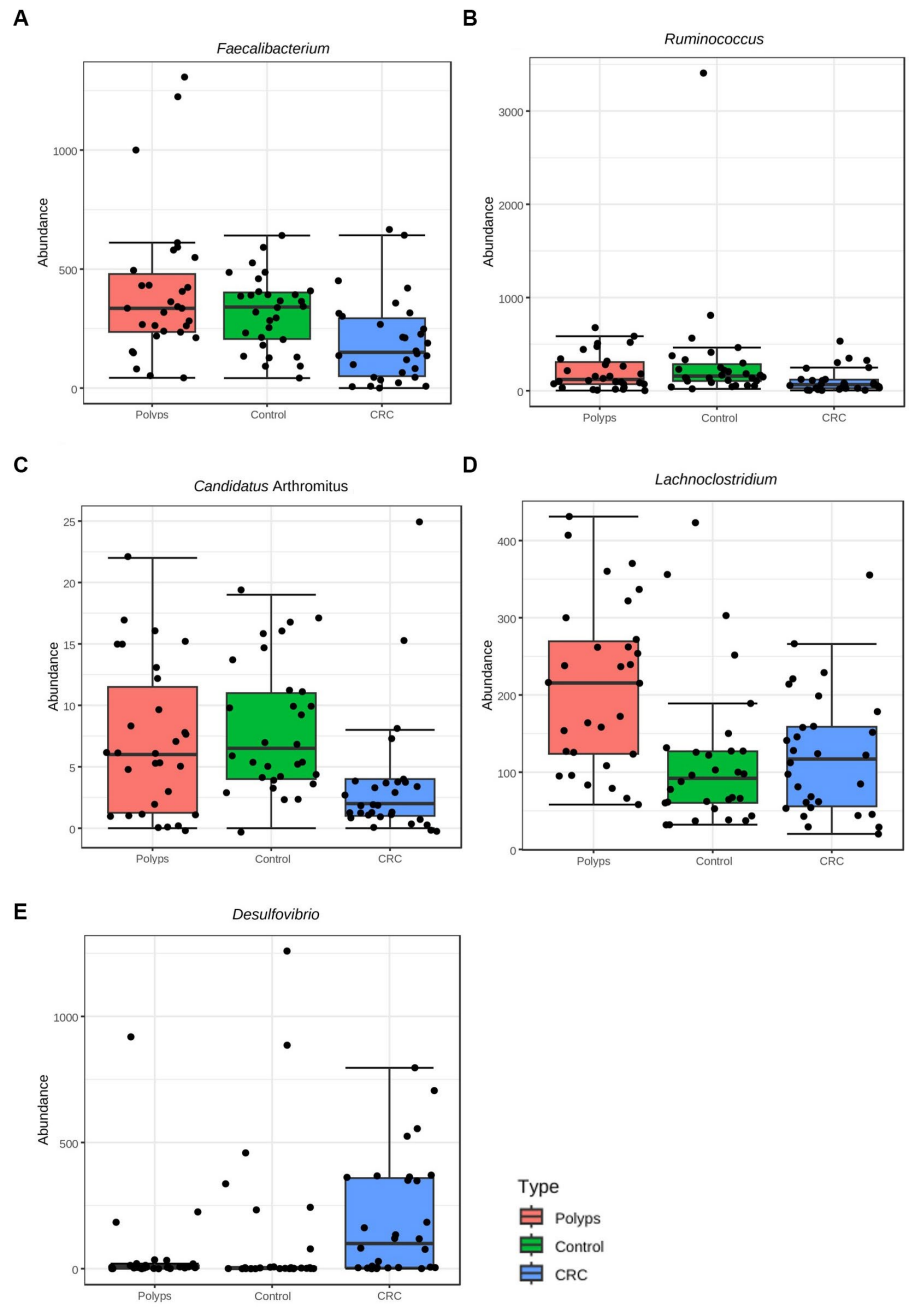
Taxa with significant differences in abundance among the three groups at the taxonomic level of Genus, using the ANOVA statistical method. **(A)** The genus *Faecalibacterium* exhibits a significant reduction in abundance in the CRC patient group (value of *p* = 0.002, FDR = 0.012), compared to the control and polyp groups. **(B)** The genus *Ruminococcus* shows a significant reduction in abundance in the CRC group (value of *p* = 0.001, FDR = 0.04), compared to the control and polyp groups. **(C)** The genus *Candidatus Arthromitus* (or Segmented Filamentous Bacteria) exhibits a significant decrease in abundance in the CRC group (value of *p* = 0.0002, FDR = 0.012), compared to the control and polyp groups. **(D)** The genus *Lachnoclostridium* demonstrates a significant increase in abundance in the polyp group (value of *p* = 0.002, FDR = 0.042), compared to the control and CRC groups. **(E)** The genus *Desulfovibrio* shows a significant increase in abundance in the CRC group (value of *p* = 0.004, FDR = 0.016), compared to the control and polyp groups.

Figure 2B illustrates the dominant genera in the three study groups (with a more detailed analysis in Supplementary material). Figure 4 highlights the genera that exhibit significant value of *p* (<0.05) and significant False Discovery Rates (FDR) (<0.05). The non-assigned genera are slightly more abundant in the CRC group (37%) compared to the control (30%) and polyp (28%) groups. *Bacteroides* is the most prevalent genus in all samples and tends to be increased in polyp patient samples (25%; control 21% CRC-18%); however, these differences are not statistically significant in our sampling context. Moreover, patients with polyps also show increased abundance, though not statistically significant, of *Parabacteroides* (4.7%) and *Barnesiela* (1.6%) compared to the control group (3.7% *Parabacteroides*; 2.5% *Barnesiela*) and CRC patients (2.9% *Parabacteroides*; 2.3% *Barnesiela*). The abundance of *Bifidobacterium*, *Dialister*, and *Akkermansia* tends to be higher in the control group (7.7%; 3.1%; 0.8%, respectively) compared to CRC (5.3, 1.5 and 0.4% respectively) and polyps (5.4, 1.6, and 0.3%, respectively), but these differences are not statistically significant in our sample condition (Figure 2B).

The representation of the genus *Ruminococcus* is significantly lower in samples from patients with CRC compared to patients in the control and polyp groups (value of *p* = 0.001; FDR = 0.04; Figure 4B; Supplementary material) *Faecalibacterium* constitutes ~1.8% of the community composition in CRC samples, and its abundance is also significantly lower compared to the control (2.7%) and polyp (2.8%) groups (ANOVA; value of *p* 0.002; FDR = 0.012) (Figures 2B, 4A). The abundance of *Suterella* and *Eubacterium rectale* is slightly reduced in CRC samples (0.84 and 0.3% respectively) compared to the control group (1.2 and 0.6%, respectively) and polyp patients (1.7 and 0.5%); however, this difference is not statistically significant (Figure 2B).

*Desulfovibrio* constitutes ~1.9% of the composition in the CRC group and is significantly overrepresented in these samples (value of *p* = 0.0004; FDR 0.016; Figure 4E) compared to the control (1.0%) and polyp (0.36%) groups (Figure 2B). On the other hand, *Prevotella* shows is slightly higher abundant in CRC samples (3%) compared to the control group (1.0%) and polyp patients (2.1%), however this difference is not statistically significant within our sample design (Figure 2B; Supplementary material).

The abundance of the genus *Lachnoclostridium* is significantly increased in the polyp’s patient group compared to the control group (1.0%) and CRC patients (1.1%) (value of *p* = 0.002; FDR = 0.042; Figure 4D). *Paraprevotella* and *Ruminiclostridium* also exhibit higher abundance in polyp patients (~0.12% for both) compared to the control group (0.8 and 0.6%, respectively) and CRC patients (0.3 and 0.4%, respectively), although these differences are not statistically significant in our sample (Figure 2B). The abundance of *Bilophila* is slightly decreased in control samples (0.8%) compared to CRC (1.3%) and polyps (1.3%), but these differences are also not statistically significant in this dataset. Lastly, the genus *Odoribacter* tends to have lower abundance in polyps (0.4%) compared to control (0.7%) and CRC (0.8%), but these differences are not statistically significant (Figure 2B; Supplementary material).

We have identified a rare taxon in our sampling that is not represented in bar chart of Figure 2B but shows a significant difference in its relative abundance among samples from different groups. This taxon was initially classified as *Candidatus Arthromitus*; however, the genus is probably classified incorrectly in the database output, as previously reported (Lundberg et al., 2017), and should be replaced by *Candidatus Savagella* or Segmented Filamentous Bacteria (SFB). *Candidatus Arthromitus* belongs to the family Lachnospiraceae (Thompson et al., 2012), while SFB belongs to the family Clostridiaceae (Thompson et al., 2013). Therefore, in this study, we interpret *Candidatus Arthromitus* as SFB (Ericsson et al., 2014; Jonsson et al., 2020). Despite its low relative abundance (0.003% in the CRC group, 0.007% in the control group, and 0.005% in the polyp group), this taxon is underrepresented in the CRC group (value of *p* = 0.0002; FDR = 0.012; Figure 4C; Supplementary material).

### 3.4 The random forest analysis indicates that *Candidatus Arthromithus* provides better classification of the CRC group, while the *Lachnoclostridium* is more effective in classifying the polyp group

**Figure 5:**
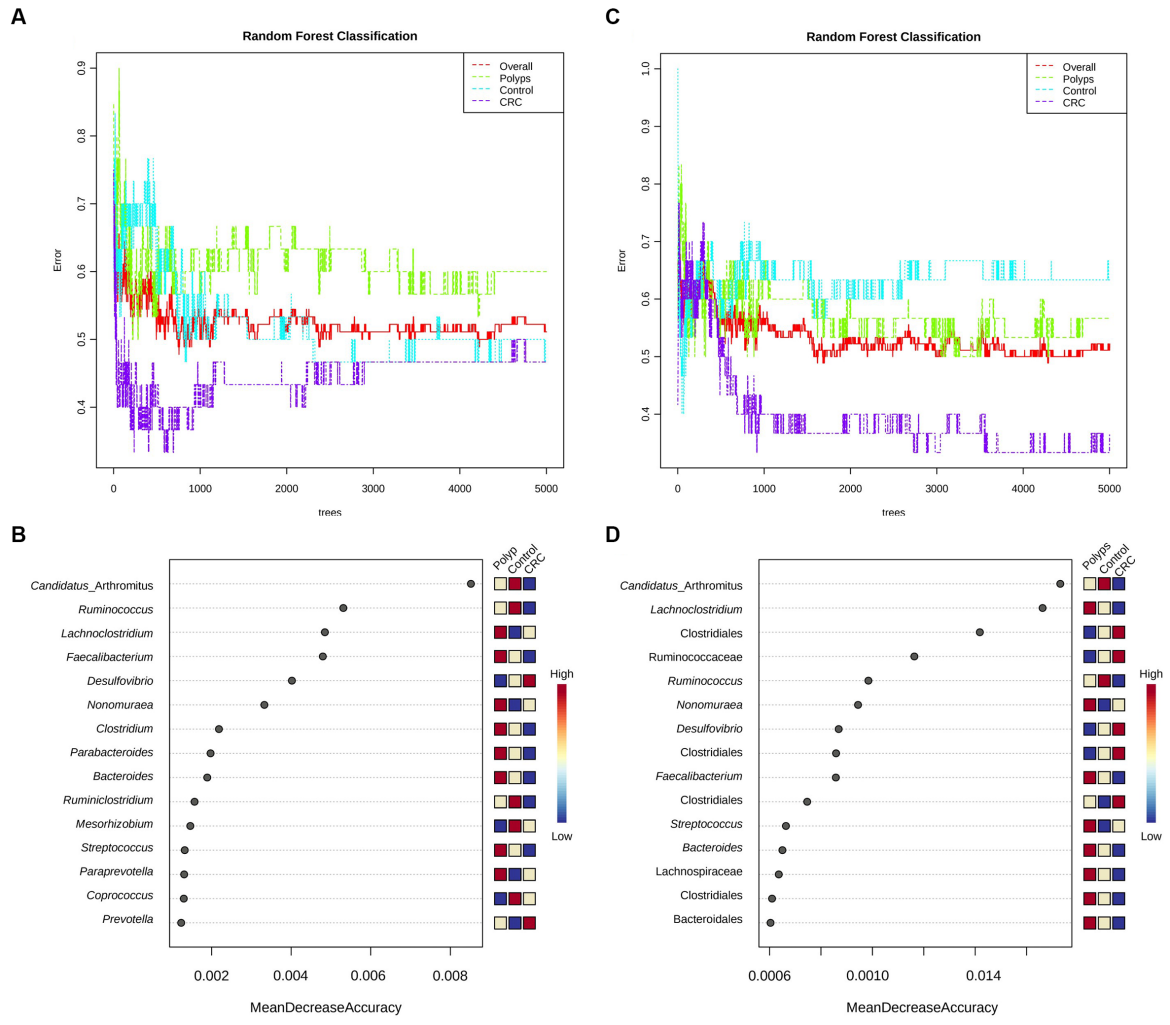
Random forest and performance analysis of classification. Both the genus-level **(A)** and OTU-level classifications **(C)** achieved significant accuracy in distinguishing the study groups; for CRC (0.367 for OTU, 0.467 for genus) and polyps (0.567 for OTU, 0.6 for genus). Out-of-bag (OOB) error rates were 0.511 for genus-based classification and 0.522 for OTU-based classification. *Candidatus Arthromitus* (or SBF) is the taxon that best classifies the three groups (precision >0.008; **B,D**) and is significantly less abundant in the samples of patients with CRC.

Random Forest analyses were conducted at the genus taxonomic level (Figure 5A) and considering the OTUs (Figure 5C) to determine which taxa would be more effective in classifying the groups. We utilized 5,000 trees and seven predictors for the analysis. At the genus level, the polyp group showed better separation than the control and CRC patient groups. For the classification based on OTUs, there was a mixture of the polyp and control groups, but better separation of the CRC patient group was observed. Both the genus and OTUs classifications achieved statistically significant accuracy in classifying the groups. Nevertheless, when considering the OTUs, the classification error rate was lower for CRC (0.367 for OTU, 0.467 for genus) and polyps (0.567 for OTU, 0.6 for genus).

Next, Figures 5B,D illustrate the classification performance of the three groups based on genus and OTUs. The *Y*-axis represents the ten most important classification variables, while the *X*-axis depicts the MDA (mean decrease in accuracy), which measures the impact on accuracy if the classifier on the *Y*-axis is removed from the classification process. At the genus level (Figure 5B), *Candidatus Arthromitus* (or “SBF”) emerges as the taxon that most effectively classifies the three study groups. It is notably less abundant in the samples from CRC patients, with an accuracy greater than 0.008. Following *Candidatus Arthromitus*, *Ruminococcus* (accuracy >0.004) is also underrepresented in the CRC group. Conversely, *Lachnoclostridium* is overrepresented in the polyp group, exhibiting an accuracy greater than 0.04. *Faecalibacterium*, with an accuracy greater than 0.004, appears to be underrepresented as well.

When considering the OTUs for classification (Figure 5D), *Candidatus Arthromitus* remains the taxon that most accurately characterizes the CRC group (accuracy >0.0014), and its abundance is significantly reduced within this group. Conversely, the abundance of the putative family Ruminococcaceae is notably increased in the CRC group, and it classifies the group with accuracy greater than 0.0010. Similarly, the genus *Desulfovibrio* achieves an accuracy of approximately 0.0010 in describing the CRC group. The genus *Lachnoclostridium* is overrepresented in the polyp patient group and exhibits a classification accuracy greater than 0.0014. Furthermore, an OTU associated with *Faecalibacterium* displays decreased abundance in the CRC patient group and provides an accuracy of approximately 0.0010 to describe this group. Two putative classes of Clostridiales demonstrate increased abundance in the CRC group, both with an accuracy exceeding 0.0006 in characterizing this group of patients.

We employed evaluation metrics for the classification model generated by Random Forest, with metrics calculated through machine learning (Table 4; see Materials and Methods). The classification based on the Random Forest data shows a slightly better precision for CRC and Polyps groups. These results indicate that our 16S rDNA data can classify the groups with moderate precision. Intragroup variations in the microbiome likely explain the observed classification precision.

**Table 4:** Evaluation metrics for the classification model generated by random forest.

Metric	Polyp	Control	CRC
Precision	64.81%	56.66%	64.56%
Recall (TPR)	43.33%	50.00%	39.47%
F1 score	51.94%	53.12%	48.99%
Accuracy	0.599%	0.559%	0.589%

### 3.5 The underrepresented genera in the CRC microbiome, *Faecalibacterium*, *Ruminococcus*, and *Candidatus Arthromitus*, demonstrate a positive co-occurrence, while the overrepresented genus *Desulfovibrio* in the CRC microbiome exhibits a negative co-occurrence with *Faecalibacterium*

**Figure 6:**
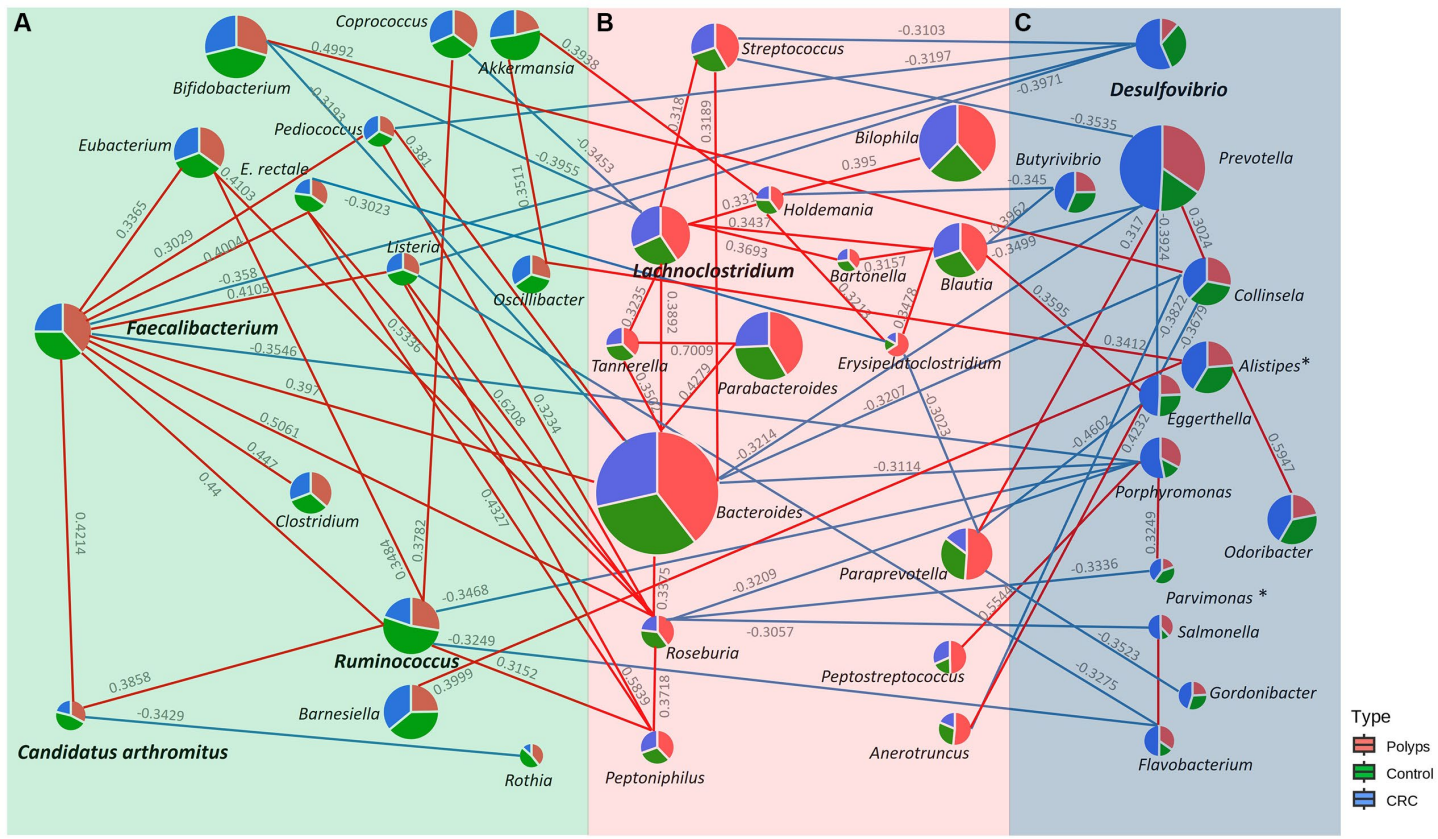
Co-occurrence network of genera with significantly increased or decreased abundance in the **(A)** Control, **(B)** Polyp, and **(C)** CRC groups. Genera with significantly increased or decreased abundance in the study groups are shown in bold, and positive and negative correlations with the main associated taxa are represented in red and blue, respectively. *Faecalibacterium*, *C*. *Arthromitus*, and *Ruminococcus*, genera underrepresented in CRC samples, positively co-occur with each other. *Faecalibacterium* exhibits a negative co-occurrence with *Desulfovibrio*, the only genus with significantly enriched abundance in the CRC group.

Figure 6 illustrates the positive (in red) and negative (in blue) correlations among the genera with significantly increased or decreased abundance in the three groups of this study, as well the main associated taxa (value of *p* <0.05; Supplementary material). The co-occurrence values are represented by the Pearson correlation coefficient, and taxa with a value of *p* less than 0.05 are considered significant. The genera *Faecalibacterium*, *C. arthromitus*, and *Ruminococcus*, which exhibited decreased abundance in the CRC group, demonstrate positive co-occurrence with each other. Additionally, *Faecalibacterium* displays a negative co-occurrence with *Desulfovibrio* (−0.358), the only genus significantly more abundant in the CRC patient group. Furthermore, *Desulfovibrio* shows negative co-occurrences with *Streptococcus* (−0.3103), *Pediococcus* (−0.3197), and *Listeria* (−0.3971). Another negative co-occurrence relationship involves *Faecalibacterium* with *Porphyromonas* (−0.3546), the latter displaying slightly increased abundance in the CRC patient samples. *Porphyromonas* exhibited positive co-occurrence with *Parvimonas* (0.3249) and *Peptostreptococcus* (0.5544), as well as negative co-occurrence with *Bacteroides* (−0.3314), *Roseburia* (−0.3209), and *Ruminococcus* (−0.3468). Moreover, *Ruminococcus* showed negative co-occurrence with *Flavobacterium* (−0.3249) and positive co-occurrence with *Coprococcus* (0.3782), *Eubacterium* (0.3484), and *Peptoniphilus* (0.3152).

*Lachnoclostridium*, the taxon that better describes the group of patients with polyps, demonstrates negative co-occurrences with *Coprococcus* (−0.3453) and *Bifidobacterium* (−0.3787). In contrast, *Lachnoclostidium* exhibits positive co-occurrence with *Bacteroides* (0.3892), *Streptococcus* (0.318), *Holdemania* (0.331), *Tannerella* (0.3235), *Blautia* (0.3437), and *Bartonella* (0.3693). *Blautia*, which is modestly overrepresented in CRC, exhibited positive co-occurrences with *Eggerthella* (0.3595) and negative co-occurrences with *Prevotella* (−0.3499), and *Butyrivibrio* (−0.345).

### 3.6 Clinical metadata indicates that the intestinal microbiome can be influenced by individual factors and underlying characteristics associated to polyps and CRC

**Figure 7:**
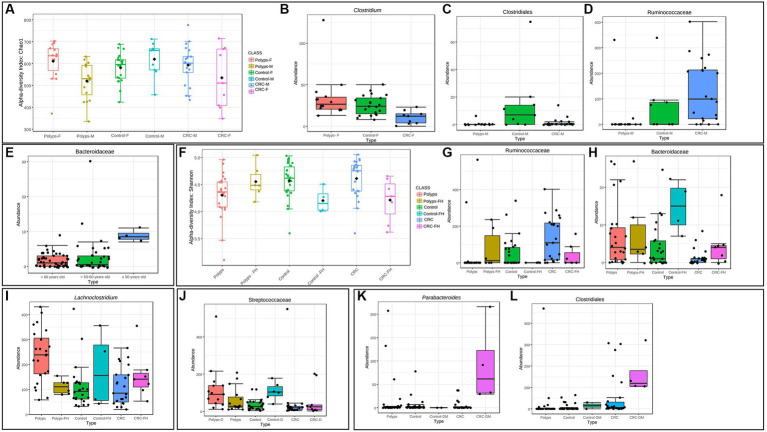
Clinical metadata that can influence the gut microbiome in polyps and CRC patients. **(A)** Influence of sex on the alpha diversity of the intestinal microbiome in patients with polyps, control, and CRC. Considering the OTUs, the diversity in the CRC and polyp groups was significantly different for this variable, being lower and higher in women, respectively (Chao 1 index; value of *p* = 0.02). **(B)** The genus *Clostridium* exhibits a significantly decreased abundance in women with CRC, compared to the control and polyp groups (value of *p* = 0.0001; FDR = 0.01). **(C,D)** Taxa that show significantly altered abundance in men. The putative Class Clostridiales is underrepresented in men with polyps and CRC, compared to the control group (value of *p* = 0.0001; FDR = 0.04). The putative Family Ruminoccoccaceae is overrepresented in the CRC group (value of *p* = 0.0001; FDR = 0.04). **(E)** The family Bacteroidaceae exhibits a significantly reduced abundance in individuals aged 50 years or older (value of *p* = 3.85E^−6^; FDR = 0.003). **(F)** Influence of family history on the alpha diversity of the gut microbiome in patients with polyps, control, and CRC. At the genus taxonomic level, the alpha diversity in the CRC and control groups with a family history of CRC was significantly lower (Shannon index; value of *p* = 0.009). **(G,H)** Taxa that show significantly altered abundance in the presence of a family history of CRC for all three groups considering the OTUs. The putative Family Ruminoccoccaceae is underrepresented in the control and CRC groups with a family history of CRC (value of *p* = 2.50E^−5^; FDR = 0.02). On the other hand, the Family Bacteroidaceae appeared with increased abundance in the control and CRC groups that had a family history of CRC (value of *p* = 9.19E^−5^; FDR = 0.02). **(I)** The genus *Lachnoclostridium* exhibits a significantly higher abundance in individuals with polyps and no associated family history of CRC (value of *p* = 0.0005; FDR = 0.03). **(J)** The family Streptococcaceae exhibits a significantly higher abundance in individuals with dyslipidemia and both polyps and control groups (value of *p* = 7.45E^−5^; FDR = 0.005). **(K,L)** Taxa with significantly increased abundance in the presence of diabetes. *Parabacteroides* and Clostridiales are increased in samples from patients with CRC associated with the clinical condition of diabetes with the use of metformin (value of *p* = 1.30E^−5^; FDR = 0.008/value of *p* = 1.91E^−5^; FDR = 0.008, respectively).

**Figure 8:**
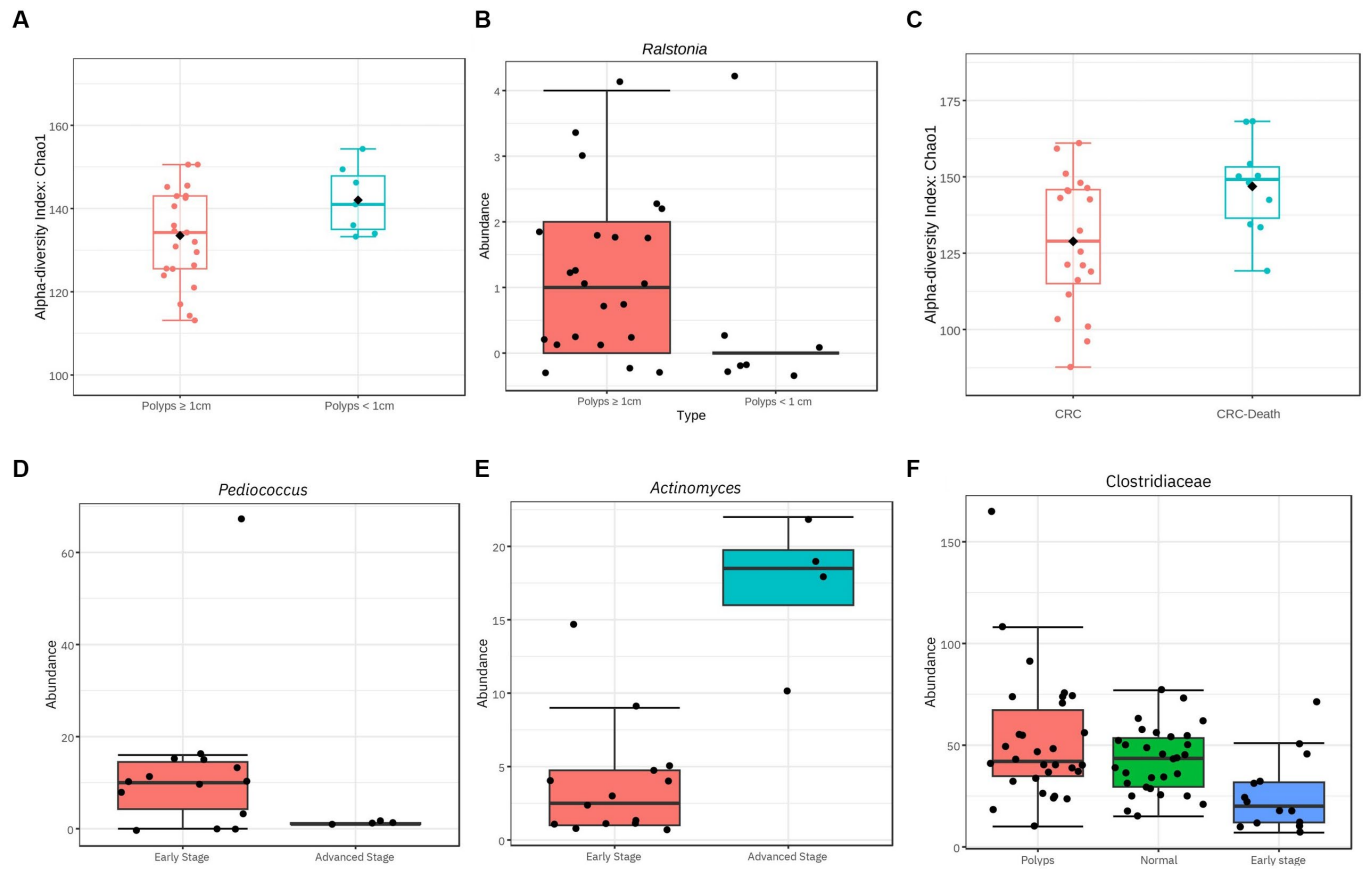
Significant differences of diversity related to characteristics of polyps and CRC. **(A)** Influence of polyp size on the alpha diversity of the gut microbiome. Considering the OTUs, polyps with a size ≥1 cm exhibit a reduction in alpha diversity (value of *p* = 0.04; Chao 1). **(B)** The genus *Ralstonia* exhibits a significantly higher abundance in individuals with polyps ≥1 cm compared to smaller polyps (value of *p* = 5.87E^−5^; FDR = 0.007). **(C)** Alpha diversity among patients with CRC is influenced by advanced clinical conditions that resulted in death. At the family taxonomic level, the diversity of the gut microbiome in patients who died was high even when compared to other patients with CRC (value of *p* = 0.014; Chao1). **(D,E,F)** Taxa with significantly different abundance in relation to CRC stage. The genus *Pediococcus* is underrepresented in individuals with stage III and IV CRC (value of *p* = 6.66E^−6^; FDR = 0.0008). The genus *Actinomyces* is overrepresented in samples from individuals with stage III and IV CRC (value of *p* = 3.56E^−5^; FDR = 0.002). The family Clostridiaceae exhibits significantly lower abundance in individuals with stage I and II CRC compared to the control and polyp groups (value of *p* = 0.0002; FDR = 0.01).

Host clinical variables, environmental and lifestyle factors may influence the gut microbiome composition. These variables cannot be controlled and potentially act as confounding factors when searching for taxonomic biomarkers associated with CRC and polyps, using data obtained from patients. To manage these confounding factors, we performed separate analyses utilizing all available clinical and lifestyle metadata. The analysis of bacterial abundance in relation to the collected metadata did not yield significant differences for most of the metadata variables examined (Tables 1–3; Supplementary Table S3). However, notable differences were observed for specific taxa (Figures 7, 8). For statistical analyses, only metadata groups with a minimum of four patients were considered representative and included in the analysis. This approach allowed us to identify taxa that had statistical differences of abundance across specific metadata categories.

In our data, it was observed that CRC was more prevalent among males, with male patients accounting for 70% of the total CRC patients (21 out of 30). However, when comparing patients in subgroups divided by sex, statistically significant differences were not found at any taxonomic level. Nonetheless, significant changes in alpha diversity were detected across all groups, both at the genus level and when considering the OTUs (Figure 7A). Alpha diversity of the CRC and polyp groups differed significantly between sexes. Men with CRC exhibited significant differences between sexes. Men with CRC displayed higher alpha diversity compared to women with CRC, while women with polyps exhibited increased alpha diversity compared to men (Chao 1, value of *p* = 0.02). Analyzing the three groups with patients separated solely by sex, similar taxonomic patterns emerged, but without statistically significant differences. Furthermore, within the female patient samples, it was found that the Clostridiaceae and the *Clostridium* genus were significantly underrepresented in the CRC group (value of *p* = 0.0001; FDR = 0.01; Figure 7B). Within the male patient samples, the abundance of the putative Clostridiales class was significantly decreased in both polyp and CRC samples (value of *p* = 0.0001; FDR = 0.04; Figure 7C), while the putative Ruminoccoccaceae was overrepresented in CRC and underrepresented in polyps (value of *p* = 0.0001; FDR = 0.04; Figure 7D).

In our sampling, the mean age was higher in the CRC group (Table 1). However, no significant differences in alpha diversity were found for this variable. Nonetheless, the putative Bacteroidaceae exhibits significantly reduced abundance in individuals aged 50 years or older (value of *p* = 3.85e^−06^; FDR = 0.003; Figure 7E).

Regarding familiar history, 19% (*n* = 17/90) of the patients reported a family history of CRC (Table 1). Control and CRC patients with familiar history of CRC exhibited significantly lower alpha diversity at genus level (Shannon index, value of *p* = 0.009; Figure 7F). However, this pattern was not observed in patients with polyps. Moreover, Ruminicoccaceae was significantly underrepresented in control and CRC patients with family history of CRC (Figure 7G, value of *p* = 2.50e^−05^; FDR = 0.02), while Bacteroidaceae is overrepresented in these patients (Figure 7H, value of *p* = 9.19e^−05^; FDR = 0.02).

Patients with polyps have a significant increase in the abundance of *Lachnoclostridium* only when there is no familiar history of CRC (Figure 7I; value of *p* = 0.0005; FDR = 0.03). Furthermore, patients with polyps showed the highest prevalence of dyslipidemia, with 50% of the group affected (*x*^2^ test, value of *p* = 0.0464; Table 1). When the three groups of interest were divided into subgroups with and without dyslipidemia, it was found that the family Streptococcaceae was significantly overrepresented in samples from patients with this comorbidity (Figure 7J; value of *p* = 7.45e^−05^; FDR = 0.005). However, in the case of patients with CRC, this taxon was not affected by the association with dyslipidemia. It is worth noting that the family Streptococcaceae was also overrepresented in the polyp group (Figure 3C).

In our sample, the most prevalent polyp subtype based on histology is tubular adenoma (Table 2). The hyperplastic subtype of serrated polyps appears in observed in almost half of the patients, often in association with other types of polyps. Polyps with a size of 1 cm or larger account for 80% of all patients. Additionally, it was found that alpha diversity at genus level is reduced in patients with polyps ≥1 cm, compared to patients with polyps <1 cm (Figure 8A; value of *p* = 0.04). Furthermore, the abundance of the genus *Ralstonia* is significantly increased in samples from patients with polyps ≥1 cm (Figure 8B; value of *p* = 5.87e^−05^; FDR = 0.007), although with low read count.

In terms of tumor localization, the majority were described in the sigmoid colon (43%) and rectum (29%), with only two patients having tumors located in the ascending colon (Table 3). However, no significant differences were found regarding tumor location in our analysis.

Regarding the staging of CRC based on the TNM classification system, our findings were limited to data from 18 out of 30 patients. Half of them were in stage I and approximately 30% were in stage II (Table 3). No taxon was associated with disease progression when analyzing all four stages together. However, when the samples were recategorized into early stages (I + II) and advanced stages (III + IV), some significant associations were observed. The abundance of the genus *Pediococcus* was significantly decreased in advanced cases of CRC (Figure 8D), while the abundance of Actinomyces was increased (Figure 8E).

Furthermore, when analyzing the 14 patients in the early stage of CRC (I + II) separately, it was found the family Clostridiaceae, previously described as underrepresented in the CRC patient group, was even less abundant (value of *p* = 0.0002; FDR = 0.01; Figure 8F). This result suggests that the abundance of the Clostridiaceae family may serve as a potential marker for early stages of CRC.

The mortality rate of the patients with CRC in this study was 33% (Table 3). Among the deceased patients, three had tumors in their rectum, two in the sigmoid colon, three in the descending colon, and two in the ascending colon. We observed a significant increase in alpha diversity at the family level in the group that progressed to death (value of *p* = 0.014; Chao1; Figure 8C). This finding suggests that patients with terminal stages of CRC may undergo an expansion of bacterial diversity in the intestinal microbiome.

In the CRC group, four patients reported having diabetes and using metformin. Interestingly, we found that the putative genus *Parabacteroides* and the class Clostridiales were significantly overrepresented in this subgroup with diabetes (Figure 7K; value of *p* = 1.30e^-0.5^; FDR = 0.008/Figure 7L; value of *p* = 1.91e^−05^; FDR = 0.008). Other comorbidities and clinical information did not show significant differences in this study, likely due to the low number of patients included in the analyses.

## 4 Discussion

In this study, we used shotgun metagenomics data to investigate bacterial signatures in the progression of CRC in a Spanish population. Our findings revealed a decrease in the alpha diversity of gut microbiota, which aligns with various intestinal and non-intestinal diseases (Malla et al., 2019). However, our alpha-diversity analysis using the Chao1 and Shannon indexes yielded contrasting results (Figures 1A,B). While Chao1 index indicated significantly lower alpha diversity in patients with polyps and CRC compared to controls, the Shannon’s index suggested a higher diversity of gut bacteria in patients with CRC. These results imply that the bacteriome of patients with CRC and polyps has experienced a loss of taxa compared to the control group, particularly those with low abundance. Nonetheless, the bacterial community of the CRC patient group exhibited greater evenness among the samples. Additionally, the NMDS analysis of beta diversity also revealed distinct bacterial distributions among the different groups (Figure 1C), indicating an altered gut bacterial composition in the presence of polyps and CRC. These findings have implications for disease diagnosis and prevention, and further investigation is warranted to understand the overlapping microbial communities and their potential role in microbial succession and response to stress factors. It is important to consider the influence of enterotype and variable microbial signatures associated with CRC in interpreting the microbiome composition of CRC patients (Yang et al., 2019a; Zhao et al., 2021).

The study identified significant taxonomic associations among patients with CRC and polyps. Moreover, the Random Forest analysis achieved a moderate level of precision in classifying the three study groups. We were able to highlight the primary taxa exhibiting statistically significant variations in abundance across these groups. However, we acknowledge the potential impact of intra-group variation on the precision of any attempt to produce a classification method. One noteworthy finding in this study was the significant difference in abundance of Clostridiaceae, particularly among patients in the early stages of CRC (Figures 3B, 8F). This observation is consistent with a previous study that reported a gradual decrease in the abundance of this family in CRC patients (Wang et al., 2017). The order Lactobacillales, which includes lactic acid-producing bacteria (LAB) known for their protective role against colorectal tumorigenesis (Bartley et al., 2018), was found to be underrepresented in the feces of CRC patients (Figure 3A). Conversely, the putative Ruminococcaceae exhibited higher abundance in the CRC group but lower representation in samples from patients with polyps (Figure 3D). These patterns were consistent with previous studies (Peters et al., 2016; Yang et al., 2019b; Yan et al., 2020). Additionally, the genus *Faecalibacterium*, associated with butyrate production (Lopez-Siles et al., 2017) which has several anti-tumor effects (Geng et al., 2021), was underrepresented in CRC patient samples (Figure 4A). The genus *Ruminococcus* also showed underrepresented in CRC samples (Figure 4B), while the putative family Ruminococcaceae displayed a significant increase (Figure 3D). The literature reports conflicting results regarding the abundance of *Ruminococcus* in the presence of CRC (Flemer et al., 2017; Ternes et al., 2020; Siddiqui et al., 2022), which may be attributed to differences in the phylogeny of these taxa families (La Reau et al., 2016) or the presence of pathogenic/opportunistic species within the Ruminococcaceae that could contribute to colorectal tumorigenesis.

*Faecalibacterium* and *Ruminococcus* exhibit positive co-occurrence with SFB (previously Candidatus *Arthromitus*), which is also underrepresented in the CRC group. These taxa form a cluster with other commensal bacteria that also produce butyrate and lactic acid, as well as display anti-inflammatory and immunogenic functions (Figure 6). SFB primarily colonizes the small intestine (Chen et al., 2018) and plays important roles in modulating the immune system (Bolotin et al., 2014; Ladinsky et al., 2019), contributing to host intestinal mucosal protection (Ivanov et al., 2009) and protection against pathogens (Woo et al., 2021). Limited studies have explored the protective effect of SFB against CRC and further research is encouraged. Additionally, *Faecalibacterium* had negative co-occurrence with *Desulfovibrio* (Figure 6), a sulfate-reducing bacteria (Rey et al., 2013), suggesting a possible antagonistic relationship between these taxa. *Desulfovibrio* was significantly more abundant in the feces of the patients with CRC (Figure 4E), consistent with previous research on sporadic CRC and Lynch Syndrome patients (Guo et al., 2016; Yan et al., 2020). Certain species of *Desulfovibrio* are present in healthy populations and colonize the gut of approximately half of all humans (Rey et al., 2013; Chen Y. R. et al., 2021). Interestingly, this study found a high abundance of *Desulfovibrio* in some individuals in the control group, indicating the presence of different *Desulfovibrio* species associated with both CRC and healthy conditions. Furthermore, factors such as dietary habits may contribute to the increased prevalence of this genus in the human gut (Wang et al., 2021).

The analysis of bacterial correlations in the bacteriome of CRC patients revealed significant associations between taxa with significantly distinct abundances and specific bacteria that showed an increased tendency of abundance (Figure 6). *Faecalibacterium* and *Ruminococcus* showed negative co-occurrence with *Porphyromonas*, a bacterial genus related to intestinal inflammation (Lee et al., 2022; Sohn et al., 2022) and periodontitis, a condition known to increase the risk of colorectal adenoma (Lee et al., 2022). *Parvimonas* and *Peptostreptococcus*, oral pathogens linked to CRC occurrence and colonic adenomas (Long et al., 2019; Ternes et al., 2020; Zhao et al., 2022), have a positive co-occurrence with *Porphyromonas*. *Porphyromonas* exhibited a negative co-occurrence with *Bacteroides*, a commensal bacteria of the human gut involved in maintaining homeostasis (Lee et al., 2013) but also associated with adenomatous polyps (Chen C. et al., 2021). Interestingly, the abundance of *Bacteroides* slightly increased in our group of patients with polyps (Figure 6; Supplementary material), Furthermore, *Roseburia*, a butyrate producer, also showed a negative co-occurrence with *Porphyromonas*, and its abundance was slightly increased in the polyp group samples, which supports similar findings from another study (Uppakarn et al., 2021).

*Lachnoclostridium*, a previously established biomarker for polyps (Li et al., 2020), was significantly enriched in our polyp group without a family history of CRC, supporting its potential for early CRC detection and highlighting the involvement of possible genetic factors. The negative co-occurrence between *Lachnoclostridium* and *Coprococcus*/*Bifidobacterium*, also reported in other study (Nogal et al., 2021), along with higher prevalence of overweight/obesity in the polyp group, may explain the increased abundance of *Lachnoclostridium. Bifidobacterium*, which has been associated with decreased abundance in polyps (Dadkhah et al., 2019), plays beneficial immune roles (Tan et al., 2016) and engages in cross-feeding with the butyrate producer *Faecalibacterium* (Rios-Covian et al., 2015), We hypothesize that the imbalances in *Lachnoclostridium* and *Bifidobacterium* may influence the colonization of *Faecalibacterium* (Kim et al., 2020), which is reduced in feces of CRC patients.

Patients with polyps also showed a significantly higher prevalence of dyslipidemia (Table 1), which is known as a potential risk factor for the development of intestinal polyps. Unfavorable cholesterol profiles are more prevalent in these individuals (Passarelli and Newcomb, 2016; Xie et al., 2019). Furthermore, we found a significant increase in the abundance of the Streptococcaceae in samples related to this comorbidity (Figure 3C), and the genus *Streptococcus* has previously been associated with the consumption of cholesterol-rich diets (Garcia-Mantrana et al., 2018). However, it is worth noting that this taxon is not affected by the presence of dyslipidemia or CRC. Polyps ≥1 cm, known as advanced adenomas (Rex et al., 2017), exhibited significantly lower alpha diversity compared to the subgroup with polyps <1 cm (Figure 8A). We propose that the loss of commensal taxa involved in maintaining colonic homeostasis may contribute to the persistence of the polyp environment and subsequent CRC development. Furthermore, we observed a significant increase in the abundance of the pro-inflammatory genus *Ralstonia*, albeit with low read counts in the samples (Figure 8B). This Gram-negative, non-fermenting aerobic genus, belonging to the phylum Proteobacteria, has been found in colonic crypts of healthy individuals (Saffarian et al., 2019) and positively associated with colorectal adenomas (Wang et al., 2022). Therefore, *Ralstonia* has the potential to enhance polyp screening in individuals with advanced adenomas, alongside *Lachnoclostridium*.

Our study revealed heterogeneity in the bacteriome of CRC patients, specially related to the diversity and taxonomic composition. Although factors such as the location and stage of CRC are known to potentially influence the variability of the microbiomes (Sheng et al., 2019; Suga et al., 2022), we did not observe associations in our study population, which may be due to the limited number of patients. Regarding the stage of CRC, the genus *Pediococcus* was significantly reduced in advanced cases (Figure 8D), and showed a negative co-occurrence with *Desulfovibrio*, suggesting a potential protective role against CRC progression (Figure 6). *Pediococcus* species have shown anti-tumoral properties against CRC (Villarante et al., 2011; Dubey et al., 2016). Therefore, further investigation is warranted to understand the potential protective role of *Pediococcus* in CRC progression. On the other hand, *Actinomyces* exhibited increased abundance (Figure 8E) and has been associated with the microbiome of patients with early-onset CRC (Xu et al., 2022), and its influence on tumor microenvironment modulation. The precise role of Actinomyces in CRC development requires further investigation. The prognosis of patients with CRC has also been linked to the microbiome (Wei et al., 2016), and we have observed an increase in alpha diversity in patients who experienced disease progression leading to death (Figure 8C). This rise in microbial diversity in advanced stages may be attributed to opportunistic pathogens not typically found in a healthy bacteriome. Furthermore, CRC patients exhibited higher relative abundance of non-assigned bacteria (Figure 2B), potentially contributing to increased diversity in advanced cases. Therefore, there is still potential for discovering new taxa that may be associated with the presence of CRC and could serve as prognostic biomarkers.

The gut bacteriome is influenced by various factors such as gender, age, family history and comorbidities (Liang et al., 2015; de la Cuesta-Zuluaga et al., 2019; Zhang et al., 2021; Ghosh et al., 2022). Our study revealed gender-associated patterns in alpha diversity measures, which support similar results in CRC samples from females (Liao et al., 2021) and findings suggest specific taxa playing important roles in CRC development in different sexes. We also observed age-related alterations in specific bacterial families, associations between the abundance of certain taxa with family history, as well as variations in bacteria associated with diabetes in individuals using metformin. However, limited sample sizes may have affected the detection of significant associations. These findings highlight the complexity of the microbiota and emphasize the need for larger studies to better understand its relationship with these factors in the context of CRC.

This study aimed to investigate the differences in bacteriome composition among control patients, individuals with polyps, and those with CRC. Our findings revealed a reduction in taxonomic richness in individuals with polyps and CRC compared to control patients, along with significant changes in the abundance of certain taxa. We identified potential taxonomic biomarkers for polyps (Streptococcaceae, *Lachnoclostridium*, and *Ralstonia*) and for CRC (Lactobacillales, Clostridiaceae, *Desulfovibrio*, SFB, *Ruminococcus*, and *Faecalibacterium*). In the early stages of CRC, the abundance of Clostridiaceae was significantly lower. We also observed a positive co-occurrence between *Faecalibacterium* and other underrepresented genera in CRC (SFB and *Ruminococcus*), while showing a negative co-occurrence with *Desulfovibrio*, indicating a potential antagonistic relationship. Furthermore, we identified heterogeneity in the bacteriome associated with polyps and CRC, with differences in taxonomic richness and/or abundance related to polyp size, tumor stage, dyslipidemia, diabetes with metformin use, sex, age, and family history of CRC. These results suggest that host clinical variables and underlying characteristics of polypoid lesions and tumors may influence the bacteriome composition, potentially acting as confounding factors in the search for taxonomic biomarkers. Our findings underscore the significance of bacteriome alterations in the occurrence and progression of CRC, while providing potential new biomarkers for early CRC diagnosis.

## Data availability statement

The datasets presented in this study can be found in online repositories. The names of the repository/repositories and accession number(s) can be found below: https://www.ncbi.nlm.nih.gov/, https://www.ncbi.nlm.nih.gov/bioproject/PRJNA961076.

## Ethics statement

The studies involving humans were approved by Biodonostia Health Research Institute/Donostia University Hospital Ethics Committee. The studies were conducted in accordance with the local legislation and institutional requirements. The participants provided their written informed consent to participate in this study.

## Author contributions

BG: Data curation, Formal analysis, Methodology, Visualization, Writing – original draft, Writing – review & editing. PF: Data curation, Formal analysis, Methodology, Visualization, Writing – original draft, Writing – review & editing. RK: Software, Validation, Visualization, Writing – original draft. GG: Software, Validation, Visualization, Writing – original draft. AV: Data curation, Formal analysis, Methodology, Writing – original draft. BJ: Writing – original draft, Writing – review & editing. AD: Writing – original draft, Writing – review & editing. KG-E: Writing – original draft, Writing – review & editing. BB: Funding acquisition, Resources, Writing – review & editing. VA: Funding acquisition, Resources, Writing – review & editing. LB: Conceptualization, Project administration, Writing – original draft, Writing – review & editing. JB: Conceptualization, Project administration, Writing – original draft, Writing – review & editing. AG-N: Conceptualization, Funding acquisition, Project administration, Resources, Writing – original draft, Writing – review & editing.
