# Supplementary material for: Identification of taxonomic changes in the fecal bacteriome associated with colorectal polyps and cancer: potential biomarkers for early diagnosis
Source: Front Microbiol. 2024 Jan 11;14:1292490. doi: 10.3389/fmicb.2023.1292490 (PMC10827328; doi:10.3389/fmicb.2023.1292490)
Supplement: Supplementary file 1 [file Table_1.DOCX]

**Supplementary Table 1. Information on read filtering.**

| **#** | **Sample** | **Reads**  **(raw data)** | **Reads (filtered)** | **%** | **OTUs** | **Microbial OTUs** |
| --- | --- | --- | --- | --- | --- | --- |
| 1 | S01 | 30824792 | 30687085 | 99,55 | 24040 | 24030 |
| 2 | S02 | 24550877 | 24516026 | 99,86 | 13765 | 13764 |
| 3 | S03 | 28246387 | 28242501 | 99,99 | 11803 | 11791 |
| 4 | S04 | 32137009 | 32133103 | 99,99 | 21485 | 21472 |
| 5 | S05 | 27773703 | 27765686 | 99,97 | 15179 | 15148 |
| 6 | S06 | 27831425 | 27710339 | 99,56 | 20049 | 20035 |
| 7 | S07 | 30000138 | 29981331 | 99,94 | 21229 | 21215 |
| 8 | S08 | 29088729 | 29080772 | 99,97 | 12485 | 12476 |
| 9 | S09 | 33517858 | 33495867 | 99,93 | 24350 | 24315 |
| 10 | S10 | 31137361 | 31129341 | 99,97 | 23947 | 23937 |
| 11 | S11 | 29557744 | 29553820 | 99,99 | 19203 | 19192 |
| 12 | S12 | 27487604 | 27480268 | 99,97 | 28365 | 28353 |
| 13 | S13 | 32023821 | 32016007 | 99,98 | 21758 | 21745 |
| 14 | S14 | 26010475 | 26003753 | 99,97 | 18222 | 18203 |
| 15 | S15 | 29193542 | 29160211 | 99,89 | 21538 | 21062 |
| 16 | S16 | 29907795 | 29882410 | 99,92 | 19682 | 19644 |
| 17 | S17 | 37870884 | 37859806 | 99,97 | 19990 | 19802 |
| 18 | S18 | 20375348 | 20369274 | 99,97 | 12562 | 12544 |
| 19 | S19 | 31235825 | 30968463 | 99,14 | 19081 | 19034 |
| 20 | S20 | 25657262 | 25649225 | 99,97 | 18838 | 18832 |
| 21 | S21 | 33391389 | 33368907 | 99,93 | 15497 | 15488 |
| 22 | S22 | 25656294 | 25565635 | 99,65 | 18693 | 18664 |
| 23 | S23 | 28922340 | 28910811 | 99,96 | 15869 | 15858 |
| 24 | S24 | 22444356 | 22438979 | 99,98 | 14787 | 14674 |
| 25 | S25 | 27351674 | 27348444 | 99,99 | 10329 | 10327 |
| 26 | S26 | 26433718 | 26418414 | 99,94 | 11253 | 11243 |
| 27 | S27 | 25115027 | 25100170 | 99,94 | 6604 | 6508 |
| 28 | S28 | 28000338 | 27985768 | 99,95 | 15527 | 15510 |
| 29 | S29 | 35702522 | 35696158 | 99,98 | 26670 | 26659 |
| 30 | S30 | 29622637 | 29614872 | 99,97 | 13454 | 13383 |
| 31 | S31 | 26291937 | 26287181 | 99,98 | 15864 | 15846 |
| 32 | S32 | 19145954 | 19141544 | 99,98 | 11388 | 11375 |
| 33 | S33 | 28201782 | 28196502 | 99,98 | 12728 | 12715 |
| 34 | S34 | 23372910 | 23367925 | 99,98 | 8842 | 8835 |
| 35 | S35 | 40893889 | 40882517 | 99,97 | 17828 | 17819 |
| 36 | S36 | 24335475 | 24332901 | 99,99 | 14623 | 14614 |
| 37 | S37 | 29293739 | 29290102 | 99,99 | 12867 | 12862 |
| 38 | S38 | 22104772 | 22102161 | 99,99 | 7163 | 7159 |
| 39 | S39 | 34262668 | 34246940 | 99,95 | 13945 | 13934 |
| 40 | S40 | 22107398 | 22105518 | 99,99 | 14129 | 14100 |
| 41 | S41 | 34392846 | 34381571 | 99,97 | 13618 | 13600 |
| 42 | S42 | 29225236 | 29207642 | 99,94 | 14129 | 14100 |
| 43 | S43 | 22466023 | 22459918 | 99,97 | 9839 | 9832 |
| 44 | S44 | 28172182 | 28161909 | 99,96 | 21385 | 21371 |
| 45 | S45 | 22553200 | 22551229 | 99,99 | 11136 | 11123 |
| 46 | S46 | 21220244 | 21214111 | 99,97 | 13298 | 13287 |
| 47 | S47 | 28087853 | 28083589 | 99,98 | 22269 | 22247 |
| 48 | S48 | 21707546 | 21698681 | 99,96 | 15936 | 15921 |
| 49 | S49 | 34158677 | 34154063 | 99,99 | 21290 | 21153 |
| 50 | S50 | 23969050 | 23960032 | 99,96 | 8258 | 8253 |
| 51 | S51 | 32649416 | 32637945 | 99,96 | 18210 | 18199 |
| 52 | S52 | 22839887 | 22837763 | 99,99 | 12319 | 12308 |
| 53 | S53 | 30263066 | 30072608 | 99,37 | 28731 | 27106 |
| 54 | S54 | 18559614 | 18555840 | 99,98 | 7953 | 7942 |
| 55 | S55 | 25690950 | 25686143 | 99,98 | 12083 | 12072 |
| 56 | S56 | 21161564 | 21157714 | 99,98 | 18285 | 18266 |
| 57 | S57 | 32262818 | 32239278 | 99,93 | 10350 | 10347 |
| 58 | S58 | 19059013 | 19054489 | 99,98 | 11527 | 11517 |
| 59 | S59 | 25875799 | 25869971 | 99,98 | 10597 | 10357 |
| 60 | S60 | 19170918 | 19148674 | 99,88 | 16057 | 16043 |
| 61 | S61 | 27896553 | 27878452 | 99,94 | 12766 | 12764 |
| 62 | S62 | 24457519 | 24444482 | 99,95 | 9967 | 9955 |
| 63 | S63 | 18160532 | 17834686 | 98,21 | 7786 | 7784 |
| 64 | S64 | 30107784 | 30094167 | 99,95 | 12558 | 12555 |
| 65 | S65 | 16887384 | 16875528 | 99,93 | 9055 | 8830 |
| 66 | S66 | 26487377 | 18830113 | 71,09 | 11974 | 11963 |
| 67 | S67 | 24147868 | 24144237 | 99,98 | 9627 | 9627 |
| 68 | S68 | 30391972 | 30359061 | 99,89 | 17693 | 17551 |
| 69 | S69 | 24000954 | 23984193 | 99,93 | 15040 | 15019 |
| 70 | S70 | 25131569 | 25120174 | 99,95 | 14382 | 14369 |
| 71 | S71 | 24393988 | 24386529 | 99,97 | 13277 | 13272 |
| 72 | S72 | 27566387 | 27557094 | 99,97 | 18637 | 18630 |
| 73 | S73 | 32295607 | 32282532 | 99,96 | 21932 | 21906 |
| 74 | S74 | 33930628 | 33925205 | 99,98 | 17953 | 17893 |
| 75 | S75 | 25444606 | 25409195 | 99,86 | 9097 | 9054 |
| 76 | S76 | 25710453 | 25693505 | 99,93 | 9754 | 9749 |
| 77 | S77 | 30737938 | 30718659 | 99,94 | 14644 | 14632 |
| 78 | S78 | 25489699 | 25487306 | 99,99 | 13138 | 13133 |
| 79 | S79 | 28197258 | 28175121 | 99,92 | 20121 | 20107 |
| 80 | S80 | 28916402 | 28829023 | 99,70 | 24576 | 24544 |
| 81 | S81 | 28432981 | 28429709 | 99,99 | 16710 | 16693 |
| 82 | S82 | 29520859 | 29517239 | 99,99 | 13980 | 13963 |
| 83 | S83 | 18409249 | 18369353 | 99,78 | 13588 | 13581 |
| 84 | S84 | 37382864 | 37378127 | 99,99 | 11857 | 11807 |
| 85 | S85 | 24394711 | 24271528 | 99,50 | 14357 | 14341 |
| 86 | S86 | 21566533 | 21253197 | 98,55 | 15041 | 15016 |
| 87 | S87 | 30010960 | 30004988 | 99,98 | 13750 | 13716 |
| 88 | S88 | 22900556 | 22801738 | 99,57 | 14291 | 14279 |
| 89 | S89 | 26886474 | 26870650 | 99,94 | 18308 | 18278 |
| 90 | S90 | 39884404 | 39863934 | 99,95 | 17195 | 17170 |
