# Supplementary figures and images for: Identification of taxonomic changes in the fecal bacteriome associated with colorectal polyps and cancer: potential biomarkers for early diagnosis
Source: Front Microbiol. 2024 Jan 11;14:1292490. doi: 10.3389/fmicb.2023.1292490 (PMC10827328; doi:10.3389/fmicb.2023.1292490)

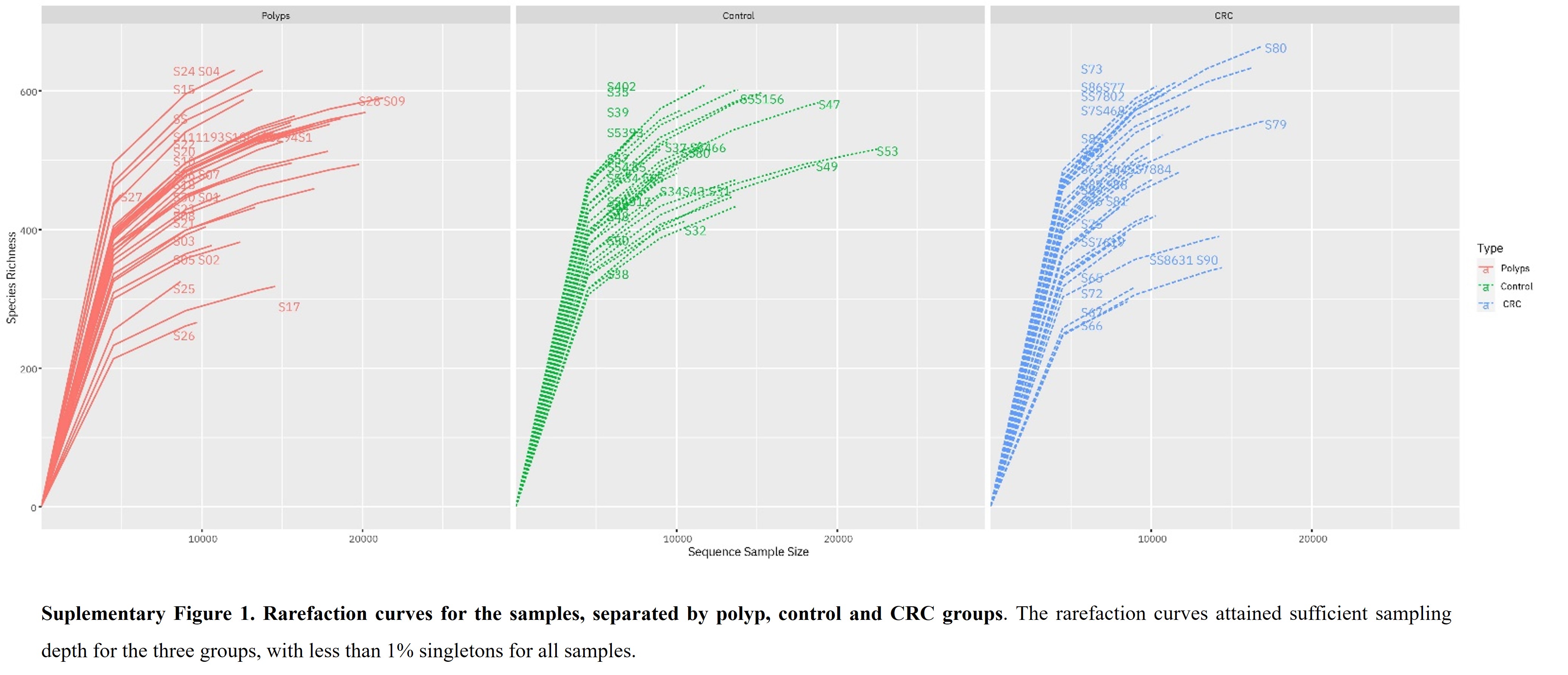

Supplement: Supplementary file 5 [file Image_1.JPEG]
